# Continental-Scale Paddy Soil Bacterial Community Structure, Function, and Biotic Interaction

**DOI:** 10.1128/mSystems.01368-20

**Published:** 2021-09-21

**Authors:** Hong-Yi Li, Hang Wang, Xin-Hua Tao, Xian-Zhe Wang, Wei-Zheng Jin, Jack A. Gilbert, Yong-Guan Zhu, Zhi-Jian Zhang

**Affiliations:** a College of Environmental and Resource Sciences, Zhejiang University, Hangzhou, China; b Yunnan Key Laboratory of Plateau Wetland Conservation, Restoration and Ecological Services, Southwest Forestry University, Kunming, China; c HangZhou GuSheng Biotechnology Co., Ltd., Hangzhou, China; d Scripps Institution of Oceanography and Department of Pediatrics, University of California—San Diego, La Jolla, California, USA; e Key Lab of Urban Environment and Health, Institute of Urban Environment, Chinese Academy of Sciences, Xiamen, China; f China Academy of West Region Development, Zhejiang University, Hangzhou, China; University of Massachusetts Medical School

**Keywords:** soil microbiota, biogeography, biogeochemical turnover, agriculture, soil microbiota

## Abstract

Rice paddy soil-associated microbiota participate in biogeochemical processes that underpin rice yield and soil sustainability, yet continental-scale biogeographic patterns of paddy soil microbiota remain elusive. The soil bacteria of four typical Chinese rice-growing regions were characterized and compared to those of nonpaddy soils. The paddy soil bacteria were significantly less diverse, with unique taxonomic and functional composition, and harbored distinct cooccurrence network topology. Both stochastic and deterministic processes shaped soil bacteria assembly, but paddy samples exhibited a stronger deterministic signature than nonpaddy samples. Compared to other environmental factors, climatic factors such as mean monthly precipitation and mean annual temperature described most of the variance in soil bacterial community structure. Cooccurrence network analysis suggests that the continental biogeographic variance in bacterial community structure was described by the competition between two mutually exclusive bacterial modules in the community. Keystone taxa identified in network models (*Anaerolineales*, *Ignavibacteriae*, and Deltaproteobacteria) were more sensitive to changes in environmental factors, leading us to conclude that environmental factors may influence paddy soil bacterial communities via these keystone taxa. Characterizing the uniqueness of bacterial community patterns in paddy soil (compared to nonpaddy soils) at continental scales is central to improving crop productivity and resilience and to sustaining agricultural soils.

**IMPORTANCE** Rice fields provide food for over half of the world’s human population. The ecology of paddy soil microbiomes is shaped by human activities, which can have a profound impact on rice yield, greenhouse gas emissions, and soil health. Investigations of the soil bacteria in four typical Chinese rice-growing regions showed that (i) soil bacterial communities maintain highly modularized species-to-species network structures; (ii) community patterns were shaped by the balance of integrated stochastic and deterministic processes, in which homogenizing selection and dispersal limitation dominate; and (iii) deterministic processes and climatic and edaphic factors influence community patterns mainly by their impact on highly connected nodes (i.e., keystone taxa) in networks. Characterizing the unique ecology of bacterial community patterns in paddy soil at a continental scale may lead to improved crop productivity and resilience, as well as sustaining agricultural soils.

## INTRODUCTION

Globally, paddy fields feed more than half of the world’s human population and are characterized by intense anthropogenic interference (1), where the relationship between human activity and rice yields is mediated by soil microbial communities, which also influence key biogeographical processes such as greenhouse gas emission (2). Previous studies have investigated paddy soil-associated microbiomes under various treatments (e.g., chemical/organic farming, varied cultivation methods, or crop rotation types), across environmental gradients over small areas, or with a small number of samples (2–4). However, our understanding of continental-scale paddy soil microbial biogeography is limited, despite the fact that paddy land covers ∼155 million ha globally (1).

The continental and global biogeography of soil microbes has been investigated previously (5–10), providing a wealth of data regarding the association between microbial community dynamics and their habitat preferences (5, 7), landscape ecological functions and services (6), and biogeochemistry and plant traits (9, 11). Most of these studies mapped microbial biogeography across natural ecosystems, but the impact of human interference as a challenge to the well-established biogeographic patterns in naturally occurring settings remains understudied (12–14). There also has been conflicting data associated with whether the microbial communities assemble through deterministic or stochastic processes (15, 16), which may be better informed through a broad-scale investigation across multiple field sites. In addition, any statistical coassociations between different paddy soil-associated microorganisms remain unclear due to the lack of large-scale studies across rice-growing regions. Exploring such associations and the potential role of interspecies interactions in shaping community structure could yield insights into the ecological assemblage of microbial communities associated with competitive niches imposed by continuous cultivation practices (17–20). This knowledge could help us understand microbial landscapes in rice-growing lands at a continental scale, significantly improving our ability to predict changes in agricultural output due to altered soil microbiota resulting from farming management practices.

We hypothesized that bacterial patterns in paddy soils would be distinctly different from naturally occurring nonpaddy soils at both regional and continental scales. We also hypothesized that, due to the selection pressure associated with paddy farming, there would be intensified biotic interactions and unique keystone taxa that greatly shaped paddy bacterial biogeographic patterns. To test these hypotheses, we characterized the paddy soil bacteria from four typical rice-growing regions in China, which together exceed 5 × 10^6^ km^2^. Paddy soils and soils from the nonpaddy habitats were collected simultaneously. The bacterial diversity, community composition, and functional potential of these soil samples were determined using 16S rRNA gene amplicon and shotgun metagenomics sequencing. Through variation partition analysis, random forest models, and cooccurrence network interpretation (21), we identified and ranked the environmental drivers and ecological processes involved in soil bacterial biogeographic patterns.

## RESULTS

### Distinct bacterial geographic patterns in paddy soils.

We collected 99 paddy soil samples and 79 nonpaddy soil samples across four typical rice-growing regions in China: Sanjiang Plain, Lianghu Plain, Taihu Plain, and Hani Terrace. The nonpaddy soil samples were mainly collected from naturally occurring wetlands, adjacent to the paddy fields. In addition to 16S rRNA amplicon sequencing analyses of these samples, 24 samples (16 from paddy soil and 8 from nonpaddy soils) were randomly selected for shotgun metagenomics sequencing.

**FIG 1:**
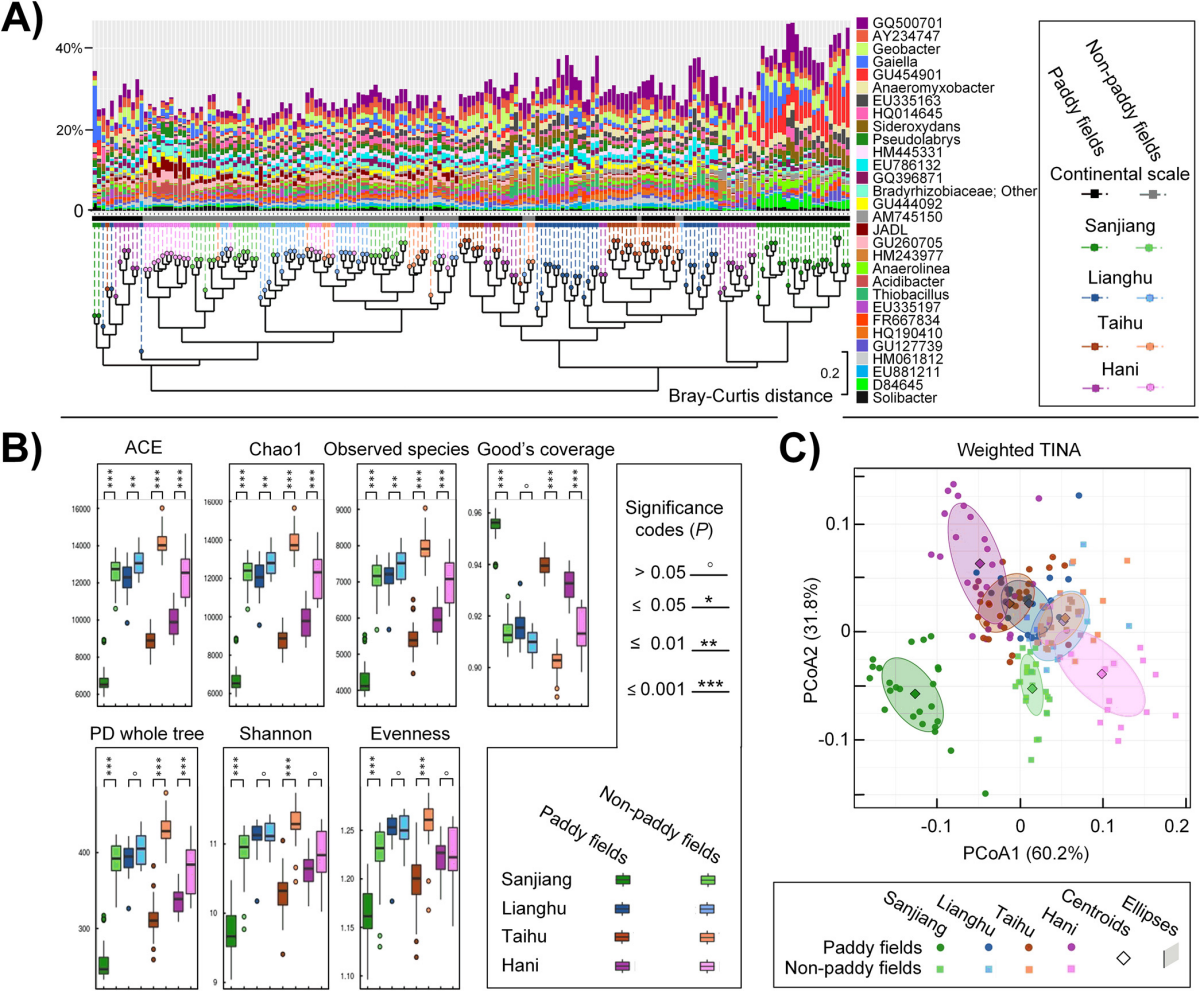
Comparison of bacterial composition and diversity in paddy and nonpaddy soils suggests the uniqueness of paddy soil-associated microbiota. (A) Relative abundance (%) of the top 30 dominant genera, clustered using UPGMA based on Bray-Curtis distances. The first line shown under the histograms is colored according to the soil type (paddy versus nonpaddy soils), and the second line is colored according to both soil type and sampling region. (B) Alpha-diversity of intraregional bacterial community, including richness (ACE, Chao1, and observed OTUs), diversity (PD whole tree and Shannon), evenness (Pielou’s evenness), and coverage (Good’s coverage). We randomly subsampled 40,000 sequences per sample 10 times to account for sequencing depth. In each boxplot, the symbols indicate the following: box, lower and upper quartiles; horizontal line, median value; and whiskers, lower and upper inner fences. Circles above or below the box plots indicate outliers. Differences between paddy and nonpaddy samples within each region were examined using a Wilcoxon rank sum test, with significance indicated above. (C) PCoA plots of samples based on the distance matrix of weighted Taxa INteraction-Adjusted index (weighted TINA index). Samples were partitioned into groups using a combination of soil type and sampling region. For each group, a filled confidence ellipse was superimposed over the data, and the added centroid (diamond) illustrates the mean distances between each pair of groups. PCoA plots based on other distance matrices are shown in Fig. S2 in the supplemental material.

We first examined whether paddy soil bacterial profiles were distinct from nonpaddy soils at both continental and regional scales. By employing UPGMA (unweighted pair-group method with arithmetic averages) based on Bray-Curtis distances between pairwise samples (Fig. 1A), we found 73 of 79 nonpaddy soil samples (92.4%) formed a cluster distinct from the paddy soil samples. Paddy soils also clustered by region, with samples from the Sanjiang Plain being the most divergent from other regions (clustering tree, Fig. 1A). Of the top 20 most prevalent microbial genera, 16 had significantly different relative abundances in paddy soils compared to nonpaddy soils, including *Geobacter*, *Anaeromyxobacte*r, *Sideroxydans*, *Pseudolabrys*, *Anaerolinea*, and *Acidibacter*, as well as a number of unidentified taxa (see Fig. S1 in the supplemental material). Some of these differences only occurred within specific regions, while others occurred across the continental scale. For example, *Anaerolinea* was enriched and *Acidibacter* was attenuated in paddy soils compared to nonpaddy soils at all sites. Using several alpha-diversity estimators, we found that paddy soils generally harbored lower bacterial richness (ACE, Chao1, and observed species), lower diversity (PD whole tree and Shannon), and reduced evenness than the nonpaddy soils in each region, with the lowest alpha-diversity found in Sanjiang Plain (Fig. 1B).

10.1128/mSystems.01368-20.3 FIG S1 Boxplots showing differential partitioning of the top 20 genera’s relative abundances. Upper panels illustrate the comparisons of paddy and nonpaddy soils across four typical rice-growing regions in China; Lower panels illustrate the comparisons of paddy soils and nonpaddy soils in each of four regions. Cross-regional and intraregional differences of each genera’s relative abundance were tested using a Wilcoxon rank sum test (*, *P < *0.05; **, *P < *0.01; ***, *P < *0.001). Download FIG S1, JPG file, 0.8 MB. Copyright © 2021 Li et al. 2021 Li et al. https://creativecommons.org/licenses/by/4.0/ This content is distributed under the terms of the Creative Commons Attribution 4.0 International license.

10.1128/mSystems.01368-20.4 FIG S2 PCoA plots of samples based on other distance matrices, including Bray-Curtis and Weighted UniFrac. For each group, a filled confidence ellipse was superimposed over the data, and the added centroid (diamond) indicates the mean distances between each pair of groups. With all indices measured, *R^2^* and *F* statistics of permutational multivariate analysis of variance (PERMANOVA) on microbial dataset were compared. Samples were partitioned into groups using a combination of soil type and sampling region. Download FIG S2, TIF file, 0.9 MB. Copyright © 2021 Li et al. 2021 Li et al. https://creativecommons.org/licenses/by/4.0/ This content is distributed under the terms of the Creative Commons Attribution 4.0 International license.

Principal coordinates analysis (PCoA) was then used to visualize gradients of bacterial beta-diversity based on classical (Bray-Curtis measures), phylogenetic (weighted UniFrac) and interaction-adjusted community dissimilarities (weighted TINA index, Taxa INteraction-Adjusted index, Fig. 1C; see also Fig. S2). Samples were found to be significantly differentiated by sampling region and soil type (paddy versus nonpaddy soils, PERMANOVA [permutational multivariate analysis of variance]; Fig. S2). The first components of PCoA based on these matrices clearly separated paddy soil samples from nonpaddy soils in each region, and both paddy and nonpaddy soils in Sanjiang Plain were obviously separated from the other sampling regions, particularly from Hani Terrace. Of all the matrices employed, the weighted TINA (Taxa INteraction-Adjusted) index provided greater explanatory power for bacterial community dissimilarity among these samples collected at continental scale (90.0% dissimilarity explained, Fig. 1C; see also Fig. S2).

**FIG 2:**
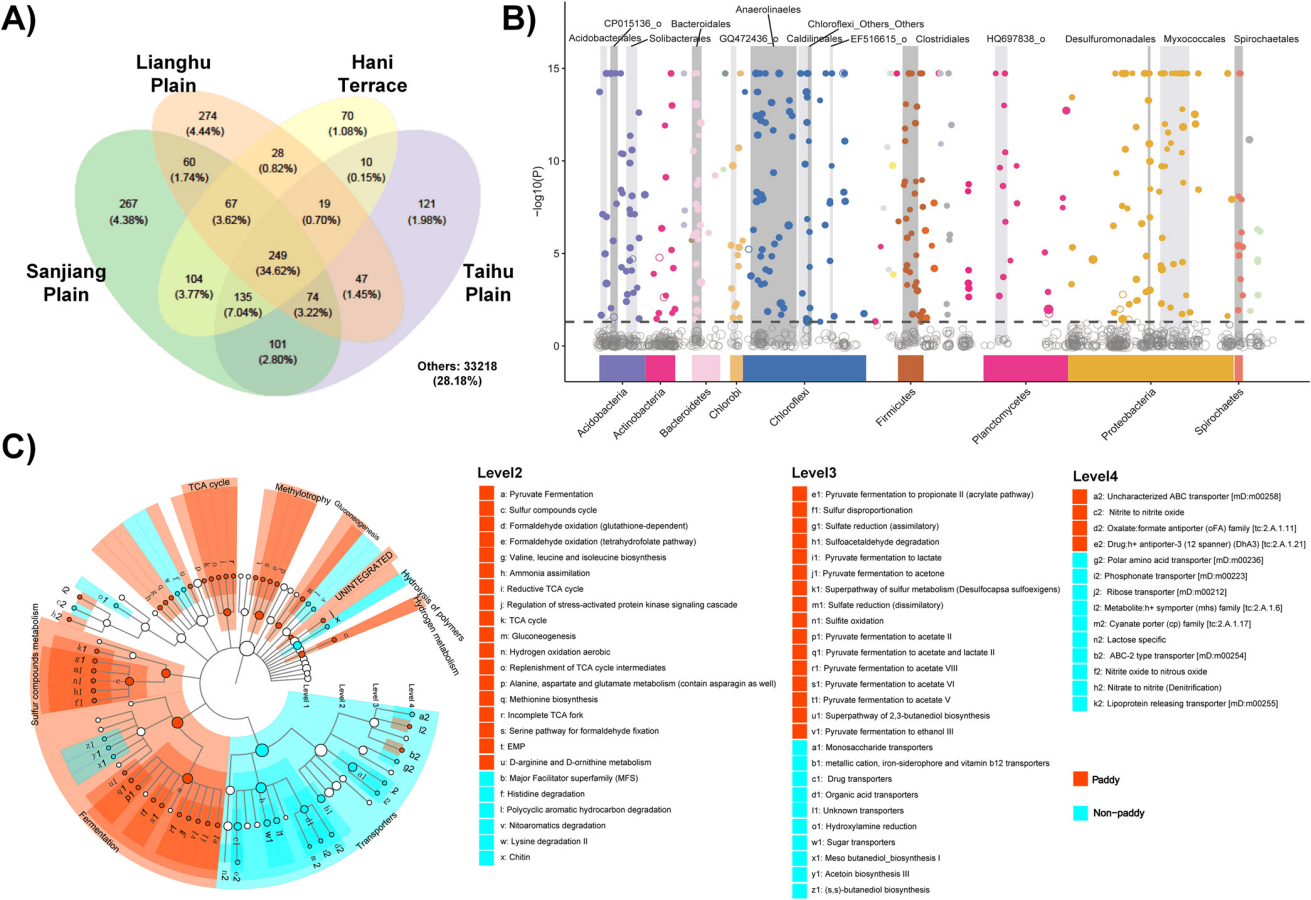
Taxonomic and functional profiling of paddy and nonpaddy soils suggests a core paddy soil microbiota with enriched functional potentials in paddy soils. (A) Overlapped bacterial OTUs among paddy soil samples in different regions using Venn diagrams. OTU numbers and their percentages of the total are shown in each segment. (B) Manhattan plot showing OTUs enriched in paddy soils compared to nonpaddy soils. Significantly enriched OTUs are depicted as full circles. The dashed line corresponds to the FDR-corrected *P* value threshold of significance (α = 0.05). The color of each dot represents the taxonomic affiliation of OTUs at the phylum level, and the sizes correspond to their relative abundances. Gray boxes are used to denote the dominant orders (with the relative abundance > 10%). The various shades of gray are used to differentiate orders, and the wider the box, the higher the relative abundance. (C) Functional differences between paddy and nonpaddy soils based on metagenomics analysis using LEfSe statistical tools. The functions were searched against a functional gene database (FOAM) and have been classified into four levels. Nonsignificantly different functions or their relative abundances <1% are not shown. Within each level, functions are listed in a decreasing order according to their relative abundances. Functions in level 1 are labeled on the clustering dendrogram.

We then used Venn diagrams to identify core-microbiota within paddy soil bacterial communities. The paddy soil core-microbiota consisted of 249 operational taxonomic units (OTUs) identified across the four regions, accounting for 34.6% of the total (Fig. 2A), and comprised predominantly *Proteobacteria*, *Acidobacteria*, and *Planctomycetes* (see Fig. S3). Through quantifying differences in taxonomic and functional characteristics between paddy and nonpaddy samples, we found that most members of these dominant phyla and their lineages (e.g., *Myxococcales*) were significantly enriched in paddy soils compared to nonpaddy soils (Fig. 2B). Paddy soils were mostly enriched in bacterial genes coding for fermentation, sulfur compounds metabolism, tricarboxylic acid cycle, methylotrophy, and gluconeogenesis compared to nonpaddy soils (Fig. 2C). Specifically, paddy soils were associated with fermentation of pyruvate to acids, energy metabolism, and sulfur and nitrogen cycling, while organic degradation and material transport was enriched in nonpaddy soils (Fig. 2C). To verify these results, the relative proportions of functional genes were also predicated from PICRUSt2 based on the taxonomic information, and we found strong agreement between those identified from the metagenomics and those predicated using PICRUSt2 (see Data Set S1).

10.1128/mSystems.01368-20.5 FIG S3 Extended information for the Venn diagram illustrated in Fig. 2A. For unique OTUs in each sampling region and the overlapped OTUs between regions, the relative abundance of OTUs and OTU occurrence frequency at both phylum (top 10%) and genus levels (top 20%) are shown. Download FIG S3, TIF file, 2.5 MB. Copyright © 2021 Li et al. 2021 Li et al. https://creativecommons.org/licenses/by/4.0/ This content is distributed under the terms of the Creative Commons Attribution 4.0 International license.

### Determinants of bacterial geographical patterns.

**FIG 3:**
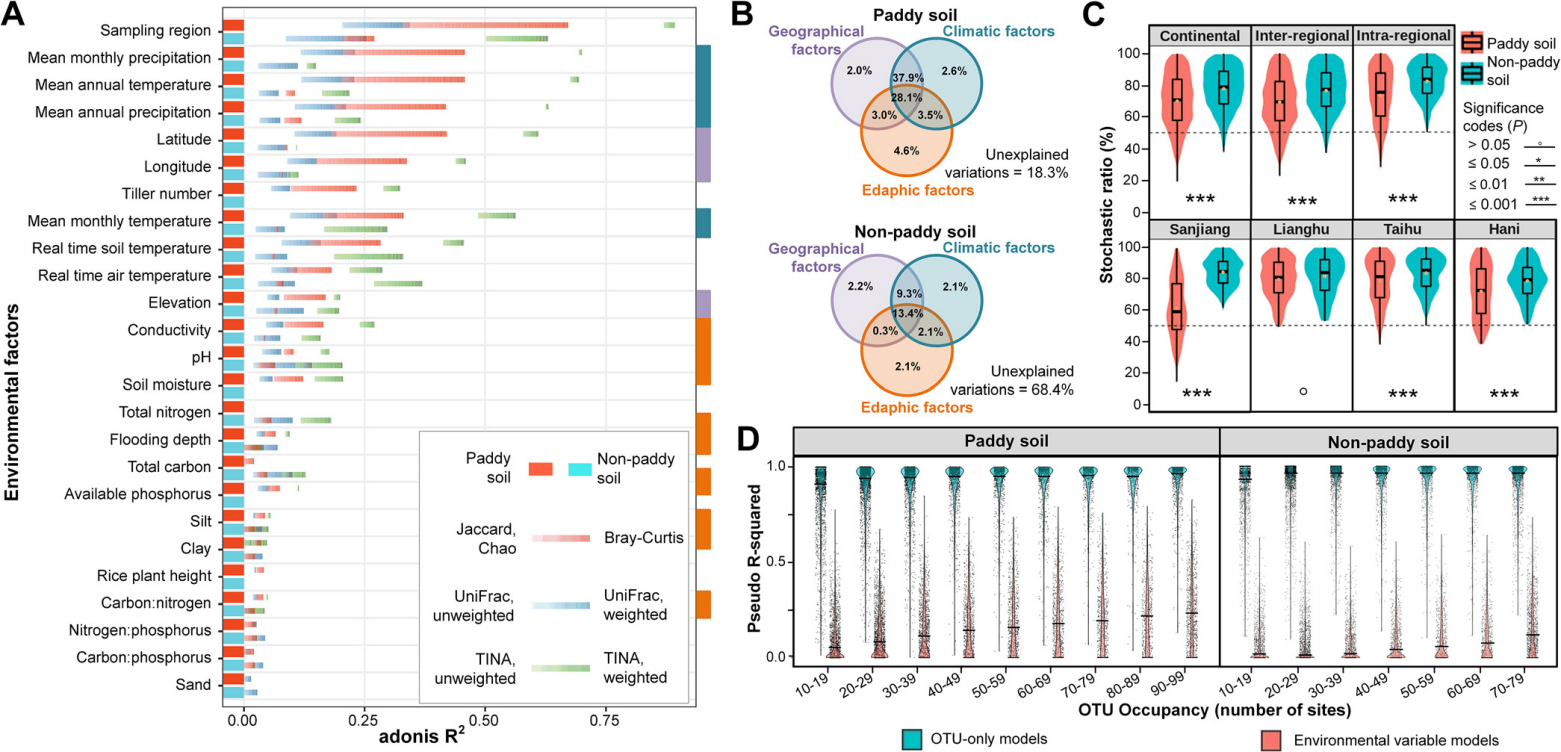
Associations between soil bacterial communities and environmental factors suggest a stronger deterministic signature in paddy samples than nonpaddy samples and also the crucial roles of biotic interactions in shaping bacterial geographic patterns. (A) Individual environmental factors as drivers of bacterial community assembly. These are listed in decreasing order following adonis *R*^2^, which explains the proportion of variance in bacterial communities. Variance in bacterial communities was assessed according to three indices (Jaccard/Bray-Curtis, UniFrac, and TINA), weighted or unweighted. (B) The influences of environmental groups (edaphic, geographical, and climatic factors) on bacterial communities were estimated using variation partition analysis. The proportion of variance explained by each group is shown. Shared effects are indicated by the overlap of circles. Factors used for variation partition analysis are indicated by color bars marked on the right side of panel A. (C) Relative importance of stochastic and deterministic processes in shaping bacterial community assembly estimated by stochasticity ratio. Boxes and whiskers indicate quartiles (10, 25, 75, and 95%), and cross-box lines indicate mean values. Differences between paddy and nonpaddy soils were examined using a Wilcoxon rank sum test. (D) Comparisons of the Pseudo-R^2^ values between two types of random forest models, predicting each OTU in bacterial assembly based on other OTUs (OTU-based) or environmental factors (environment-based). Models are grouped according to the occupancies of their targeted OTUs in paddy or nonpaddy soil samples. Violin plots show the probability density of the data using kernel density estimation. The horizontal line in each violin plot indicates the median value.

The selected environmental factors were examined as potential determinants of bacterial geographical patterns (see Data Set S2). The determination coefficients from the adonis function in the vegan package in R based on multiple matrices showed that bacterial communities were predominantly differentiated by region (sampling region, Fig. 3A) and also that mean monthly precipitation, mean annual temperature, mean annual precipitation, latitude, and longitude described the majority of the variance (adonis *R*^2^, Fig. 3A). Notably, the environmental variables measured had greater explanatory power for paddy bacterial community variance than for nonpaddy samples (TINA index; Fig. 3A). Of the multiple matrices, two novel interaction-adjusted indices, i.e., unweighted and weighted TINA indices, generally showed the highest adonis *R*^2^ values for all paddy and nonpaddy samples.

10.1128/mSystems.01368-20.9 DATA SET S1 Correlations between each FOAM ortholog from the metagenomic data, as predicted by PICRUSt2. Pearson’s correlation coefficient (cor) was used for the correlation estimation with the *P* value shown. All identified FOAM orthologs were examined. Download Data Set S1, XLSX file, 0.03 MB. Copyright © 2021 Li et al. 2021 Li et al. https://creativecommons.org/licenses/by/4.0/ This content is distributed under the terms of the Creative Commons Attribution 4.0 International license.

10.1128/mSystems.01368-20.10 DATA SET S2 Site geographic and climatic conditions and the basic physicochemical properties associated with each of the soil samples collected in this study. Download Data Set S2, XLSX file, 0.04 MB. Copyright © 2021 Li et al. 2021 Li et al. https://creativecommons.org/licenses/by/4.0/ This content is distributed under the terms of the Creative Commons Attribution 4.0 International license.

Environmental variables were then classified into three groups—edaphic, geographic, and climatic—and subjected to variation partition analysis (Fig. 3B), a method used to partition the variation of bacterial communities into fractions explained by environmental groups. For paddy samples, geographical, climatic, and edaphic factors uniquely explained 2.0, 2.6, and 4.6% of the total variation, respectively. Nonpaddy samples showed similar results, but geographic and climatic factors had greater interactions in paddy (66.0% explained) compared to nonpaddy samples (22.7% explained, Fig. 3B). To differentiate between deterministic and stochastic community assembly, the null model-based stochasticity ratios were calculated (Fig. 3C). In both soil types, stochastic and deterministic processes shaped bacterial assembly, and stochastic processes dominated (i.e., stochasticity ratio > 50%). However, paddy soils had a significantly stronger deterministic signature than nonpaddy soils (except for soils in Lianghu Plain; Fig. 3C), which was mainly imposed by homogeneous selection (a subtype of deterministic processes; Fig. S4; see also Table S1).

10.1128/mSystems.01368-20.1 TABLE S1 Relative mean contribution (%) of each scenario to the overall bacterial community assembly in paddy soils. Two indices (betaNTI and RC.bray) were determined. The betaNTI (beta nearest-taxon index) is based on a null model test of the phylogenetic diversity index betaMNTD (beta mean nearest-taxon distance), and RC.bray (modified Raup-Crick index) is based on a null model test of the Bray-Curtis taxonomic beta-diversity index. |betaNTI| < 2 indicates the community phylogenetic turnover is undifferentiated from expected turnover and the community assembly is dominated by stochastic processes. The betaNTI < −2 indicates the community assembly is determined by homogeneous selection, and betaNTI > +2 indicates variable selection. |RC.bray| < 0.95 indicates the community composition turnover is undifferentiated from expected turnover, and the community assembly is governed by ecological drift. RC.bray > +0.95 or < −0.95 indicates dispersal limitation or homogenizing dispersal, respectively. Since we had used the normalized stochasticity ratio to quantify the relative contribution of deterministic and stochastic processes, these two indices determined here were only used to quantify the subtypes within either deterministic or stochastic processes, respectively. Download Table S1, DOCX file, 0.02 MB. Copyright © 2021 Li et al. 2021 Li et al. https://creativecommons.org/licenses/by/4.0/ This content is distributed under the terms of the Creative Commons Attribution 4.0 International license.

10.1128/mSystems.01368-20.6 FIG S4 Jitter plots of betaNTI distributions for paddy soils collected from four sampling regions. (A)This analysis is a complement to the calculated stochasticity ratio shown in Fig. 3, aiming to reveal the strong homogeneous selection (betaNTI < −2) in paddy samples. (B and C) Distributions of the degree for cooccurrence network of paddy field soils (B) and nonpaddy soils (C). For comparison, Erdos-Renyi random networks are also illustrated. Download FIG S4, TIF file, 1.3 MB. Copyright © 2021 Li et al. 2021 Li et al. https://creativecommons.org/licenses/by/4.0/ This content is distributed under the terms of the Creative Commons Attribution 4.0 International license.

To investigate the role of strong biotic interactions in the geographic patterns of bacteria in our samples (as indicated in Fig. 3A), two random forest-based models were built to predict the relative proportion of each OTU in all samples using (i) other OTU proportional profiles (OTU-based model [M_OTU_]) and (ii) environmental factors (environmental variable-based model [M_ENV_]; Fig. 3D). The prediction accuracy of M_OTU_ (>95%) was significantly (Wilcoxon rank sum test, *P < *0.001) greater than that of M_ENV._

### Bacterial interactions revealed by cooccurrence network.

**FIG 4:**
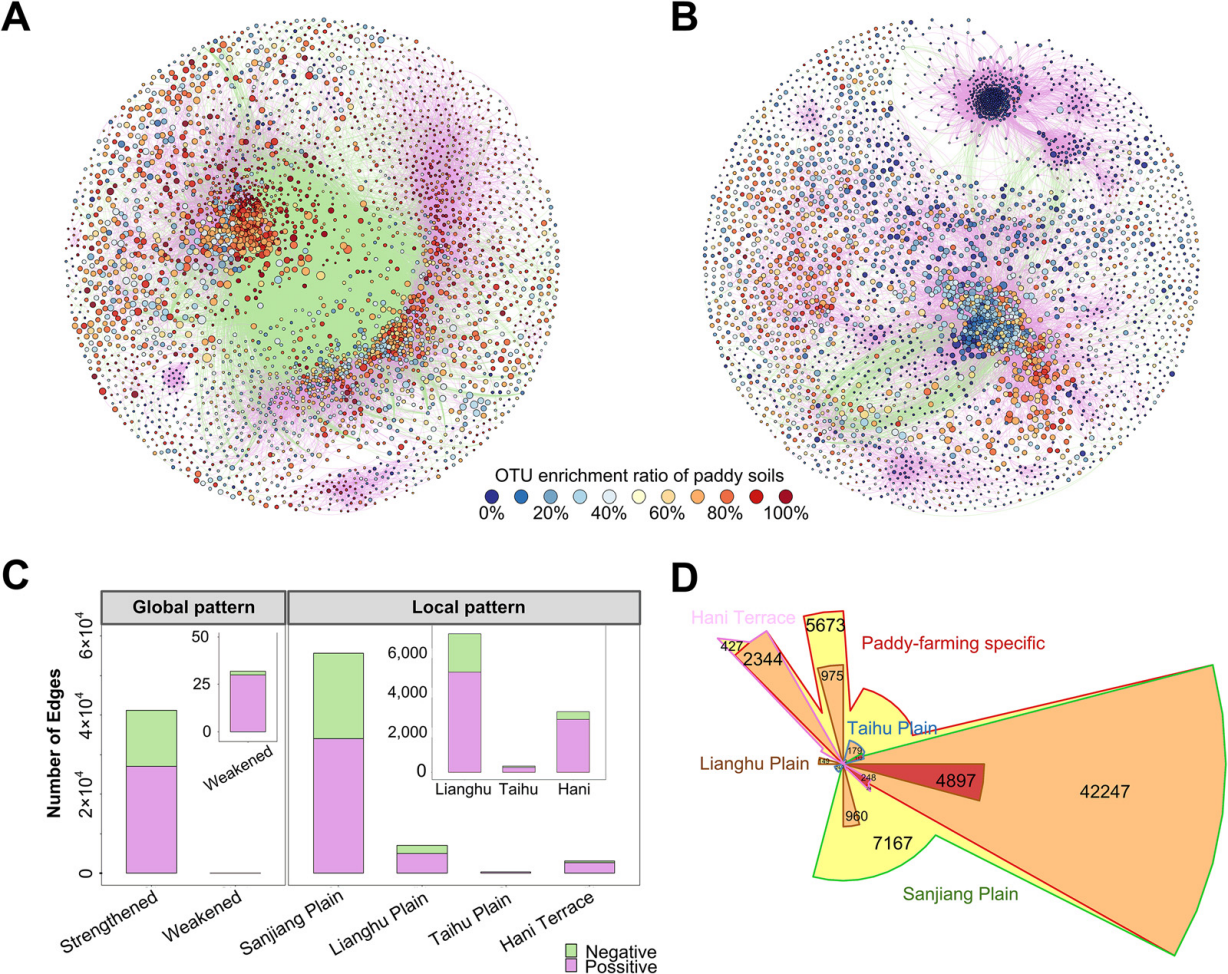
Cooccurrence networks of OTUs in paddy and nonpaddy soils across four typical rice-growing regions in China suggest strong negative associations between taxa in paddy soils globally. Cooccurrence networks visualize the different bacterial OTU interaction patterns for paddy soils (A) and nonpaddy soils (B). The nodes represent OTUs. The size of the node is proportional to the base-10 logarithm of the OTU abundance, normalized by averaging the values of each region for each soil type. Node color indicates habitat preference as revealed by OTU enrichment ratio of paddy soils to nonpaddy soils. A connection between two nodes means that there is a strong and significant (*P < *0.001) correlation between each OTU pair. The edge color indicates positive (pink) or negative (green) correlations. (C) Number of bacterial cooccurrence associations (edges) strengthened or weakened in paddy soils across four regions (left panel) and solely strengthened within each region (right panel) compared to nonpaddy soils. (D) Weighted Venn diagrams illustrating the inclusion relationships between paddy-farming specific and region-specific cooccurrence associations (edges) with the Chow-Ruskey algorithm implemented. The area is proportional to the number of the specified edges shown in each segment. Areas are colored in the order yellow, orange, and red with the increasing overlap degree.

Cooccurrence network analysis was employed to infer species-to-species associations for overwhelming effect of biotic interactions on OTU profiles in paddy soil samples. The paddy soil cooccurrence network consisted of 67,424 associations between 3,292 OTUs (Fig. 4A), while nonpaddy soil comprised 77,643 associations between 3,385 OTUs (Fig. 4B). Both networks followed scale-free degree distributions (see Fig. S4), and most OTUs captured in the networks were enriched in paddy soils (i.e., at a greater proportion) compared to nonpaddy soils across regions (Fig. 4A). Moreover, the paddy soil network had a much greater number of negative associations (antagonisms) compared to nonpaddy soil (34.4% versus 1%; Fig. 4A and B). The associations for each OTU pair involved in the paddy soil network were mainly strengthened (i.e., a higher degree of association) rather than weakened compared to nonpaddy soils (Fig. 4C). Notably, most of the associations in the paddy soil network were strengthened in Sanjiang Plain (Fig. 4C); this was verified using the Chow-Ruskey algorithm (Fig. 4D).

Different taxonomic centralities were then observed between the networks (see Fig. S5). At the order level, *Anaerolinaeles*, *Myxococcales*, *Bacteroidales*, and *Desulfuromonadales* (which were also significantly enriched in paddy soil, Fig. 2B) had the most positive (13.7, 4.9, 3.7, and 2.6%) and negative associations (13.6, 4.6, 4.4, and 3.5%) in the paddy soil networks, while their contributions to associations in the nonpaddy soils were relatively low (6.5, 3.7, 0.47, and 0.80%). In contrast, order *Planctomycetales* (7.4%) and *Rhizobiales* (6.6%) were the two most networked taxonomic groups in the nonpaddy soil network (see Fig. S5).

10.1128/mSystems.01368-20.7 FIG S5 Different taxonomic patterns (at the order level) within the cooccurrence networks of paddy soils and nonpaddy soils. (A) Circo plots showing inter-relationships of the taxonomic orders in paddy soils. (B) Circo plots showing inter-relationships of the orders in nonpaddy soils. In each circle, the different colors of segments and arcs indicate the top 39 orders, and the ribbons connecting two segments indicate copresence (left panel) and exclusion (right panel) links. For all subplots, the size of the segment is proportional to the relative abundance of the order normalized by averaging the values of each region for each soil type. The size of the arcs is proportional to OTU numbers for each order. The size of the ribbon is proportional to the number of links (copresence and exclusion), and the color is the same as to the segment with more total links (with color opacity of 0.8). Download FIG S5, TIF file, 1.1 MB. Copyright © 2021 Li et al. 2021 Li et al. https://creativecommons.org/licenses/by/4.0/ This content is distributed under the terms of the Creative Commons Attribution 4.0 International license.

**FIG 5:**
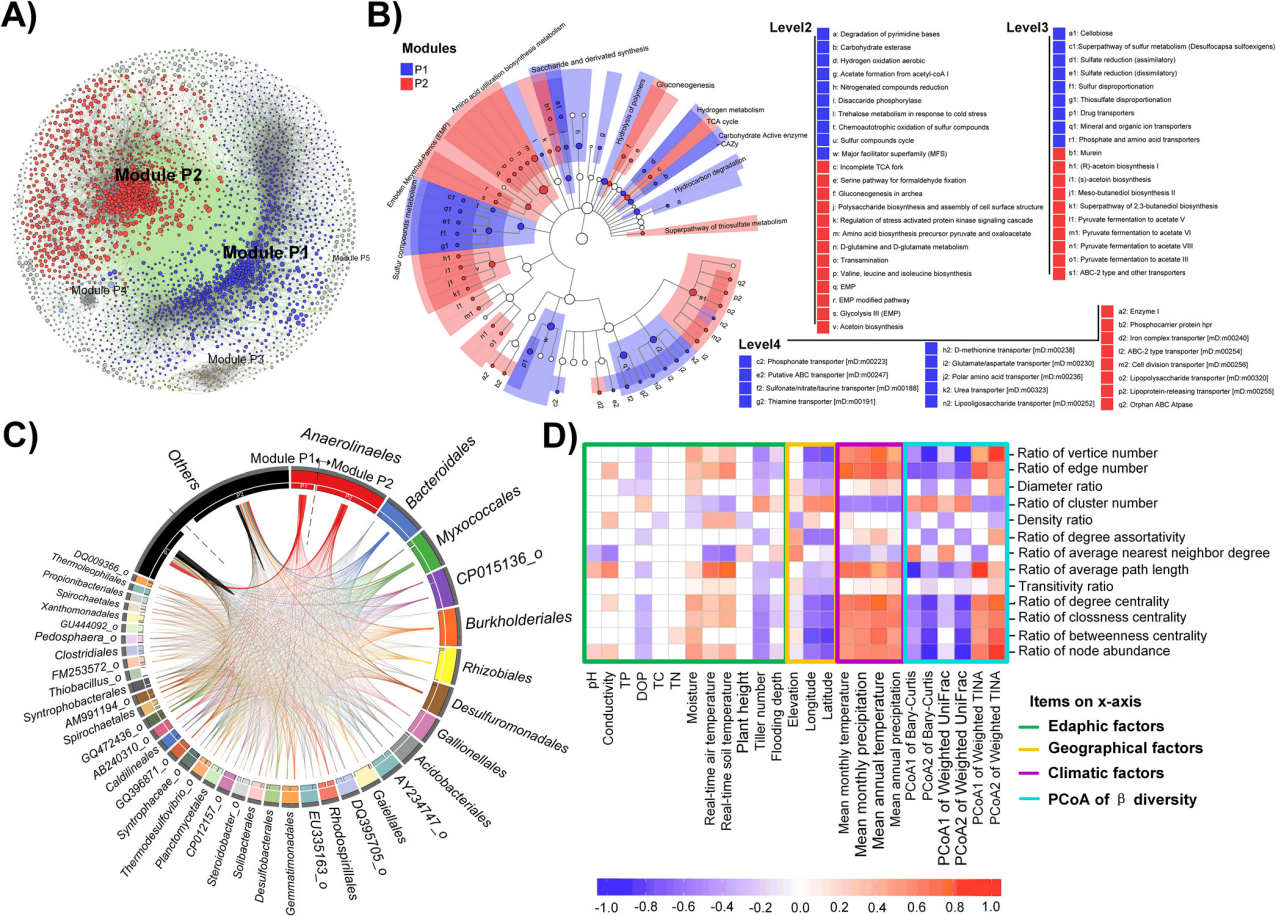
Comparison of the two major modules derived from paddy soil bacterial networks highlights their respective taxonomic members and functional potentials. (A) Cooccurrence network with nodes colored according to the top five modules (ranked by node numbers). The nodes represent OTUs. The size of the node is proportional to the base-10 logarithm of the abundance of the OTU, normalized by averaging the values of each region for each soil type. A connection between two nodes means that there is a strong and significant (*P < *0.001) correlation between each OTU pair. The edge color indicates positive (gray) or negative (green) correlations. (B) LEfSe showing functional differences between module P1 and module P2. Functions enriched in module P1 are colored in blue, and those enriched in module P2 are colored in red. The functions were searched against the FOAM database and have been classified into four levels. See Fig. 2C for details. (C) Taxonomic comparisons between modules P1 and P2 using circo plots at the order level. The size of the segment is proportional to the relative abundance of the order normalized by averaging the values of each region, and the size of the ribbon is proportional to the number of links (copresence and exclusion). The orders shown here are present in both modules. (D) Heatmap of Spearman’s rank correlation coefficients between module P1 to P2 index ratios (*y* axis) and environmental factors or the first two PCoA axes based on bacterial community composition (*x* axis).

In cooccurrence networks, a module is a cluster of highly interconnected nodes. We calculated the modularity index of the paddy (0.524) and nonpaddy soil networks (0.513), both of which suggest a modular structure (modularity index > 0.4) (17). Based on this, we grouped paddy and nonpaddy soil network nodes into 214 and 246 modules, respectively. A total of 81.29% of the nodes in the paddy network were in the first two modules (Fig. 5A), while 86.1% of the nodes of nonpaddy network were grouped into the first three modules (see Fig. S6). The first two modules of the paddy soil network (module P1, 1,557 nodes; module P2, 1,119 nodes) maintained a high degree of negative associations between nodes (Fig. 5A), while only a few OTUs in module E1 had negative associations in the nonpaddy soil network (see Fig. S6). In paddy fields, the two modules had different taxonomic member preference for community assembly. The module P1 was enriched in *Chloroflexi*, *Gemmatimonadetes*, *Chlorobi*, *Planctomycetes*, and *Latescibacteria WS3*, whereas the module P2 was enriched in *Acidobacteria*, *Actinobacteria*, *Bacteroidetes*, and *Verrucomicrobia* (*P < *0.05, Wilcoxon rank sum test; Fig. 5A). The functional gene predictions showed that Module P1 was enriched for sulfur cycle and organic biosynthesis, while module P2 was enriched for Embden-Meyerhof pathways and fermentation (Fig. 5B). *Bacteroidales*, *Anaerolinaeles*, and *Syntrophaceae* in module P2 and *Solibacterales*, *Clostridiales*, *Syntrophobacterales*, and *Myxococcales* in module P1 had the most associations (Fig. 5C, as indicated by the size of the ribbon in the circo plot). Further, in terms of topological features, there were significant correlations linking environmental factors and bacterial diversity to the difference degrees of the modules in the paddy soil network (Fig. 5D).

10.1128/mSystems.01368-20.8 FIG S6 Cooccurrence network of microbial community in nonpaddy soils. Each node is colored according to the top 3 modules (ranked by node numbers). The nodes represent OTUs. The size of the node is proportional to the base-10 logarithm of the OTU abundance, normalized by averaging the values of each region for each soil type. A connection between two nodes means that there is a strong and significant (*P < *0.001) correlation between each OTU pair. The edge color indicates positive (gray) or negative (green) correlations. Download FIG S6, JPG file, 2.9 MB. Copyright © 2021 Li et al. 2021 Li et al. https://creativecommons.org/licenses/by/4.0/ This content is distributed under the terms of the Creative Commons Attribution 4.0 International license.

### Interplay between biotic interactions and abiotic environmental effects.

**FIG 6:**
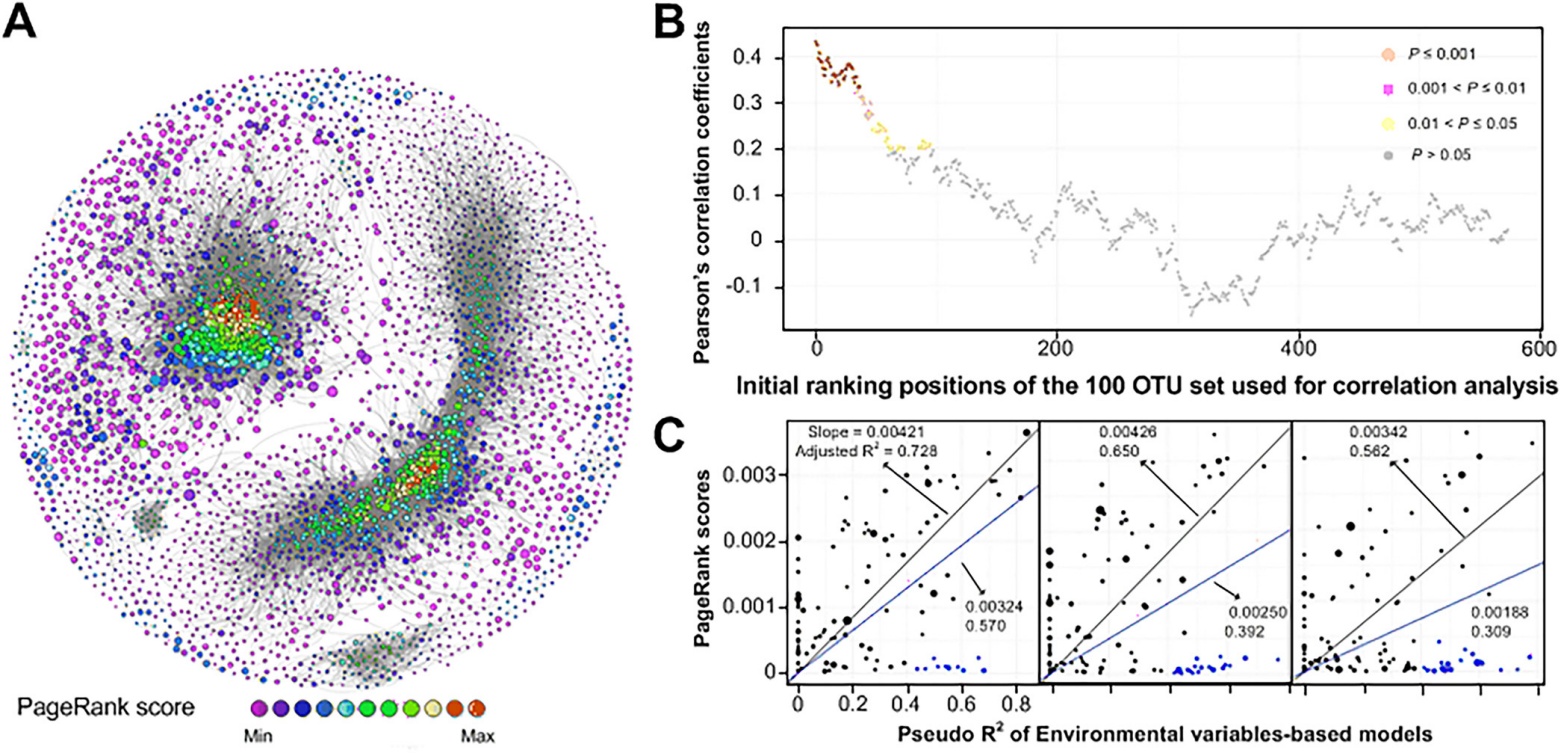
Correlation between node PageRank scores in the paddy soil bacterial network and the environmental explanatory powers indicates a strong interplay of biotic-abiotic factors. (A) Cooccurrence network of bacterial community in paddy soils, with nodes colored according to their PageRank scores, as a reflection of nodes importance in the network. The size of the node is proportional to the base-10 logarithm of the OTU abundance, normalized by averaging the values of each region for each soil type. A connection between two nodes means that there is a strong and significant (*P < *0.001) correlation between each OTU pair. The edge color indicates positive (gray) correlations. (B) Pearson’s correlation coefficients between PageRank scores and the environmental explanatory powers (Pseudo-R^2^) within 100 successive nodes with the nodes moving from 0 to 574. (C) Linear relationships between PageRank scores and the environmental Pseudo-R^2^ within node sets “1 to 100,” “51 to 150,” and “100 to 200.” The blue line is the fit line of all nodes, and the black line is the fit line of black nodes only (with the exclusion of the blue nodes). Nodes were ranked by their descending abundances. Blues nodes are identified as those distributed in the outskirts of the modules.

Since both abiotic (i.e., environmental variables) and biotic (species-to-species interactions) factors were crucial drivers of bacterial community structure in paddy soils, the abiotic-biotic interplay was further assessed. For this, keystone taxa were first identified from nodes in modularized networks, and we found that most were members of the order *Anaerolineales*, *Ignavibacteriae*, and Deltaproteobacteria (Fig. 6A; see also Table S2). These keystone taxa have been ranked according to their PageRank score values, which were used to indicate their importance in the networks according to an algorithm developed for web search engines, as previously reported (22). To test whether the environmental factors influence bacterial community structure predominantly by invoking changes in keystone taxa, as a reflection of abiotic-biotic interplay, we calculated the Pearson’s correlation coefficients between PageRank scores and the environmental explanatory powers (Fig. 6B). The most influential OTUs (as ranked by their PageRank scores) were also those most affected (*P < *0.001) by environmental factors. A positive linear relationship was shown between PageRank score and the environmental Pseudo-R^2^ (Fig. 6C). We also noticed there were some less influential OTUs (15.6%, with low PageRank scores), which could also be explained by environmental factors. By excluding these OTUs, a stronger correlation was observed (Fig. 6C).

10.1128/mSystems.01368-20.2 TABLE S2 Keystone taxa identified in modules in paddy soil bacterial network implemented with PageRank algorithm. For each keystone taxon, its pageRank score and relative abundance are shown. Download Table S2, DOCX file, 0.02 MB. Copyright © 2021 Li et al. 2021 Li et al. https://creativecommons.org/licenses/by/4.0/ This content is distributed under the terms of the Creative Commons Attribution 4.0 International license.

## DISCUSSION

We performed a continental-scale biogeographical study of paddy soil bacterial communities. Confirming previous large-scale soil studies (5, 7), paddy soil harbored a core microbiota (most of which were members of the *Proteobacteria*, *Acidobacteria*, and *Planctomycetes*, Fig. 2) and was also enriched for *Chloroflexi*, which mostly included genera of *Longilinea*, *Levilinea*, and *Anaerolinea*, all belonging to the order *Anaerolineales* (Fig. 2). *Anaerolinea* is involved in the fermentation of various sugars derived from rice plants (straw) and is promoted by hydrogenotrophic methanogens (23, 24). Paddy soil was also enriched for *Desulfuromonadaceae* (including *Geobacter* and *Anaeromyxobacter* [Fig. 2; see also Fig. S1]), which perform acetate metabolism and can compete with methanogens via dissimilatory Fe(III) reduction (25). Also enriched was the genus *Sideroxydans*, which is a chemoautotroph using iron or sulfur as energy sources (26). Harboring these enriched bacteria suggests why paddy soil metagenomes were enriched for fermentation, organic metabolism, and elemental (such as iron and sulfur) cycling genes, compared to nonpaddy soils (Fig. 2C). Notably, paddy soils were also enriched for methylotrophic-associated genes (Fig. 2). Compared to nonpaddy soils, the regular flooding-draining alternation mode of paddy field management is conducive to the formation of a characteristic oxic/anoxic soil profile, which selects for unique bacteria and functions.

Deterministic community assembly processes generally result from combined abiotic (external environmental) and biotic filtering (i.e., bacterial interactions) effects. Such processes were clearly stronger in paddy than nonpaddy soil (Fig. 3C). The extensive disturbance experienced in paddy soil may aggravate competition in bacterial communities, which would filter out species that could not tolerate local environmental change, favoring niche assembly (27). The alpha-diversity was significantly lower than for nonpaddy soils, possibly due to extreme selection pressure for specific taxa. Given uniform agricultural management practices, the reduction in site-to-site variability in soil fertility status in paddy fields (compared to nonpaddy lands) explains the poor explanatory powers of nutrients and their stoichiometry in shaping bacterial communities (Fig. 3A). Bacterial community variance driven by dissolved organic matter availability in paddy soils is regulated by precipitation (28), and in the present study the bacterial geographic patterns were strongly associated with temperature, precipitation, and pH (Fig. 3A), similar to other soil systems (29). The distinct geographic patterns in bacterial geographic distribution at the continental scale could be interpreted as being a result of strong deterministic processes found in paddy soils.

Biotic factors, such as other taxa, were important in influencing overall bacterial community dynamics in paddy soil (Fig. 3D). This was further supported by the importance of the weighted TINA index (30), which outperformed other indices in the interpretation of bacterial variation (Fig. 1C; see also Fig. S2). The explanatory powers of environmental variables on the bacterial communities were greatly enhanced when interaction signals were incorporated into community matrices by TINA (Fig. 3A), suggesting environmental variables impose strong selection on overall biological interactions, rather than upon discrete individuals. We believe that these novel insights are the first to emerge from exploring the bacterial communities of paddy soils at a continental scale.

The paddy soil network had a greater modularity and included more mutual exclusion relationships compared to the nonpaddy soil network. Continuous ploughing and waterlogged conditions could make this soil habitat more homogeneous, leading to relatively weak niche differentiation (13, 31). Alternatively, most agricultural soils such as paddy soils are continuously fertilized (31), which exposes the microbiota to superfluous exogenous substrate and unbalanced element stoichiometry. These factors may co-explain the maintenance of two network modules (i.e., module P1 and module P2) with clear negative associations between them (Fig. 5A). Microbial competition is key to both individual and community success and can regulate the assembly and maintenance of community structure (32). Therefore, we conclude that the outcomes of paddy soil bacterial biogeographic patterns are determined by a trade-off between these two modules (Fig. 5D). Interestingly, comparisons also revealed the coupled functional processes existing in two respective models. For instance, the coupled organic biosynthesis (module P2) and polymer hydrolysis/hydrocarbon degradation (module P1; Fig. 5B) indicated there were strong trade-offs of the specific function and its counterpart, mediated by the distinct bacteria in different modules. This provides evidence of potential antagonistic interactions in paddy bacterial communities; however, these biotic interactions will need to be validated, since we have only demonstrated statistical associations. Despite this, our findings provide approximations of the real interactions, and these can be assessed to predict microbiome functioning.

Central to microbial survival is the ability to acquire nutrients and energy and to interplay with environmental conditions (32). In general, our data indicate that the more influential a specific taxon is in the network, the more sensitive they are to environmental dynamics (Fig. 6). Those with the greatest influence (keystone taxa; see Table S2) may have particular environmental adaptations that support their influence. For example, taxa belonging to the *Anaerolineae* can synthesize adherence proteins that promotes cellular attachment, facilitating cell aggregation and biological interactions (33), whereas taxa from the order *Desulfobacterales* could establish redox connections with other microbial species through extracellular electron transport pathways encoded by E-pilin genes (34). These features convince the roles of these species as the keystones in real ecological circumstance. Keystone taxa have been frequently considered “ecosystem engineers,” whose removal can completely destabilize an ecosystem (20). Climate variability (especially precipitation) across geography was especially important in the assembly of bacterial communities in paddy fields at the continental scale (Fig. 3B); therefore, the environmental vulnerability of the keystone taxa across geographic regions suggests that these bacteria may be well adapted to climate variance.

The paddy soil bacterial diversity, community composition, and structure was well conserved at both regional and continental scales; this observation is likely to be associated with stronger environmental filtering processes compared to nonpaddy soils. Our data suggest that the environmental vulnerability of keystone taxa and the strong biotic-abiotic interplay mediate large-scale paddy soil bacterial dynamics. However, the bacterial interactions under highly disturbed agricultural practices require thorough investigation, since they may be central to the productivity and sustainability of this important cropping system. Overall, elucidating the uniqueness of paddy soil bacteria, their strong biotic interaction potentials, and environmental sensitivities of keystone taxa greatly advances our knowledge about microbial biogeographic patterns in well-managed agricultural ecosystems.

## MATERIALS AND METHODS

### Site selection and soil sampling.

Soil samples were collected from 99 paddy fields and 79 surrounding lands across four typical rice-growing regions of China mostly during the tillering phase of rice growth in 2015 and 2016. Of the 99 paddy soils, 24 were from Sanjiang Plain, 26 from Lianghu Plain, 26 were from Taihu Plain, and 23 were from Hani Terrace; of the 79 soils from surrounding lands, there were 23 from Sanjiang Plain, 13 from Lianghu Plain, 24 from Taihu Plain, and 19 from Hani Terrace. Four regions are different in rice cultivation history, management, and fertilization applications. In details, the rice cultivation history of Sanjiang Plain, Lianghu Plain, Taihu Plain, and Hani terrace are around 60, 5,000, 4,000, 1,200 years, respectively. Lianghu and Taihu Plain support two-season rice growth or rice-wheat rotation, while rice only grows once per year in Sanjiang Plain and Hani Terrace. The amounts of fertilizers applied are the highest in Taihu Plain (nitrogen at 190 to 300, P_2_O_5_ at 48 to 60, and K_2_O at 59 to 79 kg/hm^2^/y) and the lowest in Sanjiang (nitrogen at 90 to 165, P_2_O_5_ at 45 to 61, and K_2_O at 40 to 45 kg/hm^2^/y) according to Chinese government statistical bulletins. The soils from all paddy fields were classified as three major groups: submergenic, gleyed, and hydromorphic paddy soils (Chinese soil taxonomy). At each sampling site, five soil cores (2.5-cm diameter by 15-cm depth) were collected randomly within a certain area (5 m × 5 m, in general) and homogenized to yield a single soil sample. The sample was immediately put into sterilized plastic tubes, sealed, and placed in liquid nitrogen or in dry ice for transportation. After shipping into the laboratory, some tubes were stored at −20°C or air dried and then ground for soil physical and chemical analyses; others were stored at −80°C for DNA extraction. We collected nonpaddy soils in a similar way to the method used paddy soils. For each nonpaddy soil sample, it was collected from lands adjacent to the paddy fields in a pair. The nonpaddy soils were mostly from natural wetlands and served as potential “candidates” for the growing of rice. Based on our sampling scheme, the distance between paddy and nonpaddy lands in each pair was 100 to 500 m, depending on the local landscape conditions. Previously, we examined the molecular diversities of soil dissolved organic matter and their correlations with soil microbial communities in paddy fields, and more details about soil sampling and pretreatment can be found there (28). Environmental variables were determined according to standard methods described by Bao (35) or were obtained from local meteorological stations. These variables have been grouped into (i) edaphic variables (including soil moisture, pH, conductivity, total carbon, total nitrogen, total phosphorus, the ratio of carbon to nitrogen, the ratio of carbon to phosphorus, the ratio of nitrogen to phosphorus, available phosphorus, soil clay, silt, and sand content), (ii) geographical variables (including latitude, longitude, and elevation), and (iii) climatic variables (mean annual temperature, mean annual precipitation, mean monthly temperature, and mean monthly precipitation, real-time soil temperature, and real-time air temperature). Other variables pertaining to rice stages or conditions (including rice plant height, flooding depth, and tiller number) were also recorded.

### 16S rRNA sequencing and analysis.

Soil DNA was extracted using the MoBio PowerSoil DNA extraction kit and was normalized to the equal concentrations before downstream processing. The V4-V5 region of the 16S rRNA gene was amplified in triplicate using the F515/R907 primer set and marked with barcodes. These amplicons were then sequenced on Illumina HiSeq 2500 platform according to manufacturer’s protocol (Novogene, China). The QIIME v1.9.1 pipeline (36) was employed for the preprocessing of the raw sequence data. First, barcodes were extracted from the data set, and paired-end reads were merged to get consensus sequences with quality scores using the default setting. Then, the multilane fastq data were demultiplexed to assign sequences to samples. After stripping the primer pairs and making the 3′ ends aligned for each sequence, quality filtering was performed using the “fastq_filter” function in usearch v10.0.240 (37) with the maximum expected error threshold setting to 1.0. A total of 42,871,532 reads were pooled with >40,000 reads for each sample. Dereplication of sequences was performed using the “derep_fulllength” function in vsearch v2.3.3 (38) with the minimum abundance for output setting to 2. The nonredundant sequences were clustered into OTUs using the “cluster_otus” function in usearch with a 97% consensus threshold (39). Then, the pooled sequences were mapped to OTUs using the “usearch_global” function in vsearch with the same consensus threshold, and the unmapped sequences were removed. Taxonomy assignment was accomplished with the “parallel_assign_taxonomy_rdp.py” function in QIIME (36) in which RDP’s Classifier was used to search the EzBioCloud database (40). Notably, the database uses typical 16S rRNA sequence accession to name unclassified taxon levels. The abundance and taxonomic information for OTUs were then converted to the BIological Observation Matrix (BIOM) format for further analyses. PICRUSt2 (41) was used to predict the abundance of KEGG ortholog compositions of the bacterial communities in each sample. The resulting orthologs were then mapped to a functional gene database (FOAM), and these FOAM ontologies were classified as one of four levels to describe their functional hierarchy (42).

### Shotgun sequencing, metagenome assembly, and annotation.

Of 24 soil samples, 16 metagenomic sequencing data for paddy soil samples (4 samples from each region) were adopted from our previous study (28); 8 samples from nonpaddy soils were randomly selected for shotgun metagenome sequencing using the same protocol. Metagenome sequencing yielded about 14.0 Gb per sample after quality control. For functional annotation, metagenome assemblies were first conducted using MEGAHIT v1.1.1, which yielded a total of 41.8 M contigs >200 bases in length and 10.1 M contigs >500 bases in length. The protein translations of genes were then predicted from these contigs using prodigal with “-p meta” (43), and finally a protein catalog with pooled proteins was annotated using the FOAM database (42) in order to obtain orthologs which were defined using KEGG Orthology (44).

### Random forest-based models.

For each OTU with an occupancy not less than 10, we built two models (i.e., OTU versus other OTUs [MOTU] and environmental factors [MENV]). Cross-validation regression was performed using OTU abundance as a dependent variable and the abundance of other OTUs or selected environmental factors as independent variables (21). For each regression, up to 20 variables were selected by using the minimum Redundancy Maximum Relevance (mRMR) filter-ranking algorithm. Before data import, the continuous variable data matrix was discretized using the minimum description length principle (MDLP) algorithm. Random forest regression was followed by a leave-one-out cross-validation (using cv.fold = 99 in the function rfcv). The variable subset with the maximum Pseudo-R^2^ (1 – normalized mean square error) was selected. A paired Wilcoxon test adjusted using a Benjamini-Hochberg false discovery rate (BH-FDR) was used to test the significance of the differences between three models. R package randomForest, discretization, stats, and multtest were used for the above analyses.

### Network construction and topological feature analysis.

We initially use the derived OTUs for paddy soil samples to construct network of paddy soils. To eliminate the effect of asymmetric sample size, we chose 13 random samples per sampling region for paddy soils. We removed OTUs found in less than 9 of these samples. The abundances of these OTUs were recalculated according to the filtered new data set, while their correlation matrix was calculated based on all the 99 paddy soil samples using a custom R implementation of SparCC (45). The pseudocount value in SparCC was set to 10^−6^. Adjusted *P* values were calculated using the two-stage Benjamini and Hochberg false discovery rate (TSBH-FDR) controlling procedures with the R package multtest. Based on FDR-adjusted *P* values and correlation coefficients, we constructed cooccurrence network with nodes representing 97% cutoff OTUs and edges representing correlations between these OTUs. The cutoff of FDR-adjusted *P* values was set as 0.001. The cutoff of correlation coefficients was determined as 0.62 through random matrix theory-based methods (46) as implemented in R package RMThreshold. Using the same procedure, the network of nonpaddy soils was also inferred based on the SparCC correlation coefficients and a cutoff of 0.59. All networks were explored and visualized with the interactive platform gephi (47). The nodes in networks represent OTUs, and the edges that connect these nodes represent correlations between OTUs.

Topological features of the generated networks were first calculated with the igraph package (48). The nodes in the networks were then ranked according to their PageRank score values, which were calculated according to an algorithm from a web search engine, as previously reported (22), and can be used to indicate the importance of the nodes in the networks. Network modularity was also calculated using the gephi built-in algorithm with a resolution of 8, which describes the modularity characteristics of the network (47). The network nodes were then grouped into different modules identified from the network.

To investigate the influence of paddy management, global trends, or regional signals on the species-to-species relationships, samples were divided into groups according to paddy/nonpaddy soil and sampling region memberships. The impact of paddy farming or regional influence on the correlations value of each edge in the network was assessed by dividing the omission score (OS; the SparCC correlation value without these samples) by the absolute value of the original SparCC score. To account for group size, the OS was computed repeatedly for random and same-size sample sets. The nonparametric *P* value was calculated as the number of times that the random OS was smaller than the sample group OS, divided by the number of random OS (500 for each OTU pair). Each edge was determined as “paddy-specific” or “region-specific” when the ratio of OS to absolute original score was <1 and the TSBH-FDR adjusted *P* value was <0.05.

### Statistical analysis.

Bacterial alpha-diversity was measured by considering community richness (i.e., ACE, Chao1 and Observed OTUs), diversity (i.e., PD whole tree and Shannon), evenness (Pielou’s evenness), and coverage (Good’s coverage) using QIIME 1.9.1 (36). The beta-diversity was measured using count-based indices (i.e., Chao’s Jaccard and Bray-Curtis), phylogenetic indices (i.e., unweighted UniFrac and weighted UniFrac), and two novel interaction-adjusted indices (i.e., unweighted TINA and weighted TINA) (30). PCoA was conducted with following PERMANOVA performed using the adonis function. Variation partition analysis was used to calculate the independent influences of environmental factors grouped within different categories. All the above analyses were conducted using the R package vegan (49). Spearman’s rank correlation coefficient was used for correlation analysis of bacterial network topological features and environmental factors. Core microbiomes (defined as OTUs appeared in 100% samples) of paddy soils were analyzed using Venn diagrams. The enrichments of these OTUs compared to nonpaddy soils based on the relative abundances were analyzed using metagenomeSeq analysis and then illustrated using Manhattan plots. The differential functional potentials between bacterial communities of paddy and nonpaddy soils were further examined using LEfSe (50). Comparisons were conducted using linear discriminant analysis with a threshold of 2.5. The differential functional potentials between the two main modules of bacterial network in paddy soil were also examined using LEfSe (50) with the same parameters but an LDA threshold of 2.0. To assess the concordance between the FOAM ontologies derived from the real metagenomic data and those predicated by PICRUSt2, Pearson’s correlation coefficients were calculated. Normalized stochasticity ratio (51), betaNTI, and RC.bray (52) values were calculated to quantify the relative importance of stochastic and deterministic processes in shaping the bacterial community assembly.

### Data availability.

The raw sequencing data sets of 16S rRNA for soil samples (except for 11 paddy soil samples in Lianghu Plain) have been deposited in sequence read archive at NCBI under project accession no. PRJNA573301. The raw data of metagenomics sequencing have been deposited under project accession no. PRJNA385547 (for selected paddy soils) and PRJNA576996 (for selected nonpaddy soils). Of our data set analyzed here, 11 of the raw 16S rRNA data for paddy soil samples from Lianghu Plain can be found under PRJNA385062 (i.e., SRR5500614, SRR5500616, SRR5500617, SRR5500619, SRR5500621, SRR5500623, SRR5500624, and SRR5500626 to SRR5500629). All other data needed to evaluate the conclusions in the paper are present in the paper and/or in the supplemental material. Correspondence should be addressed to Y.-G.Z. and Z.-J.Z. Requests for additional materials and database should be addressed to H.-Y.L. (1025597656@qq.com) and H.W. (hwang17@163.com).
