# Supplementary figures and images for: Continental-Scale Paddy Soil Bacterial Community Structure, Function, and Biotic Interaction
Source: mSystems. 2021 Sep 21;6(5):e01368-20. doi: 10.1128/mSystems.01368-20 (PMC8547477; doi:10.1128/mSystems.01368-20)

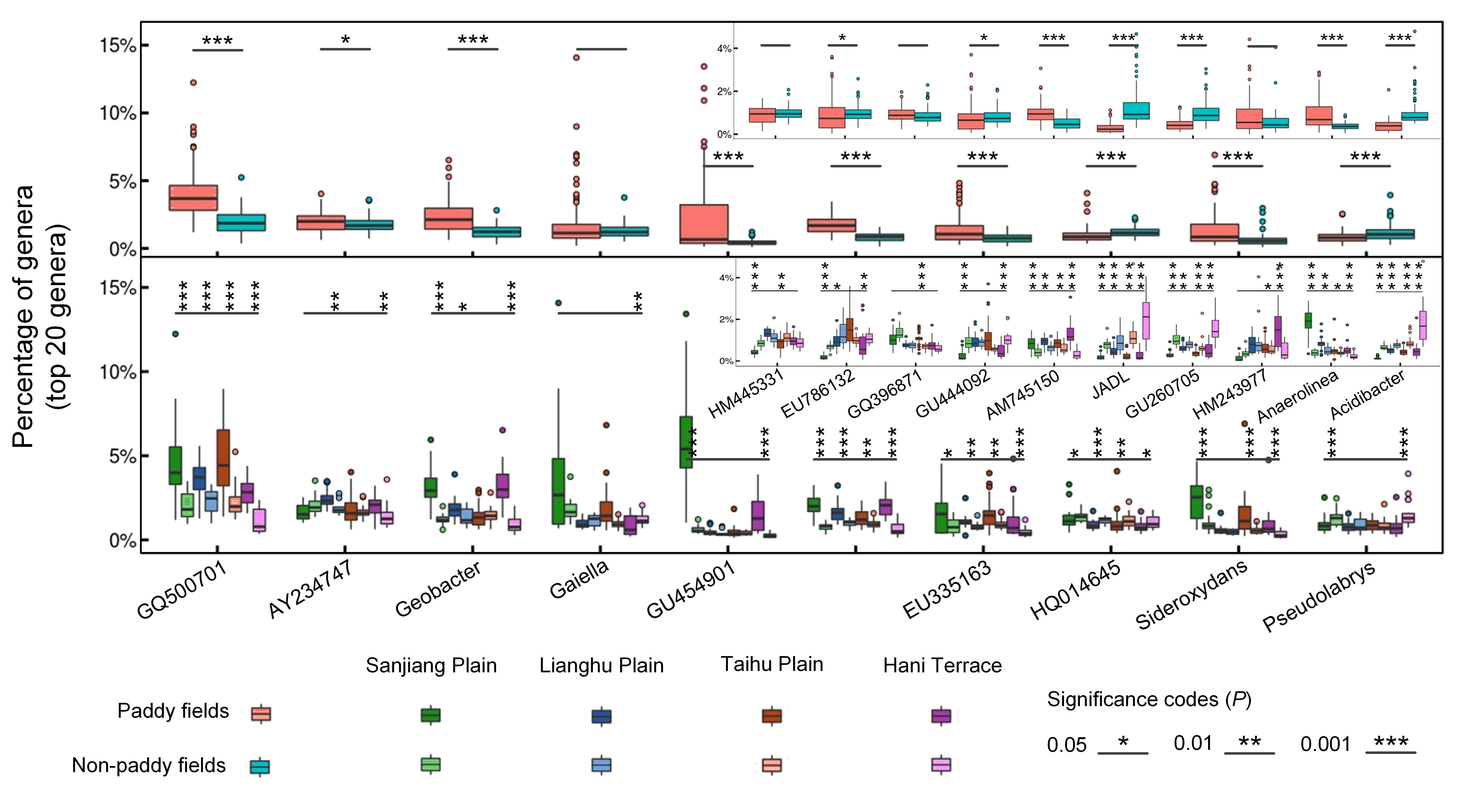

Supplement: FIG S1 [file msystems.01368-20-sf001.jpg]

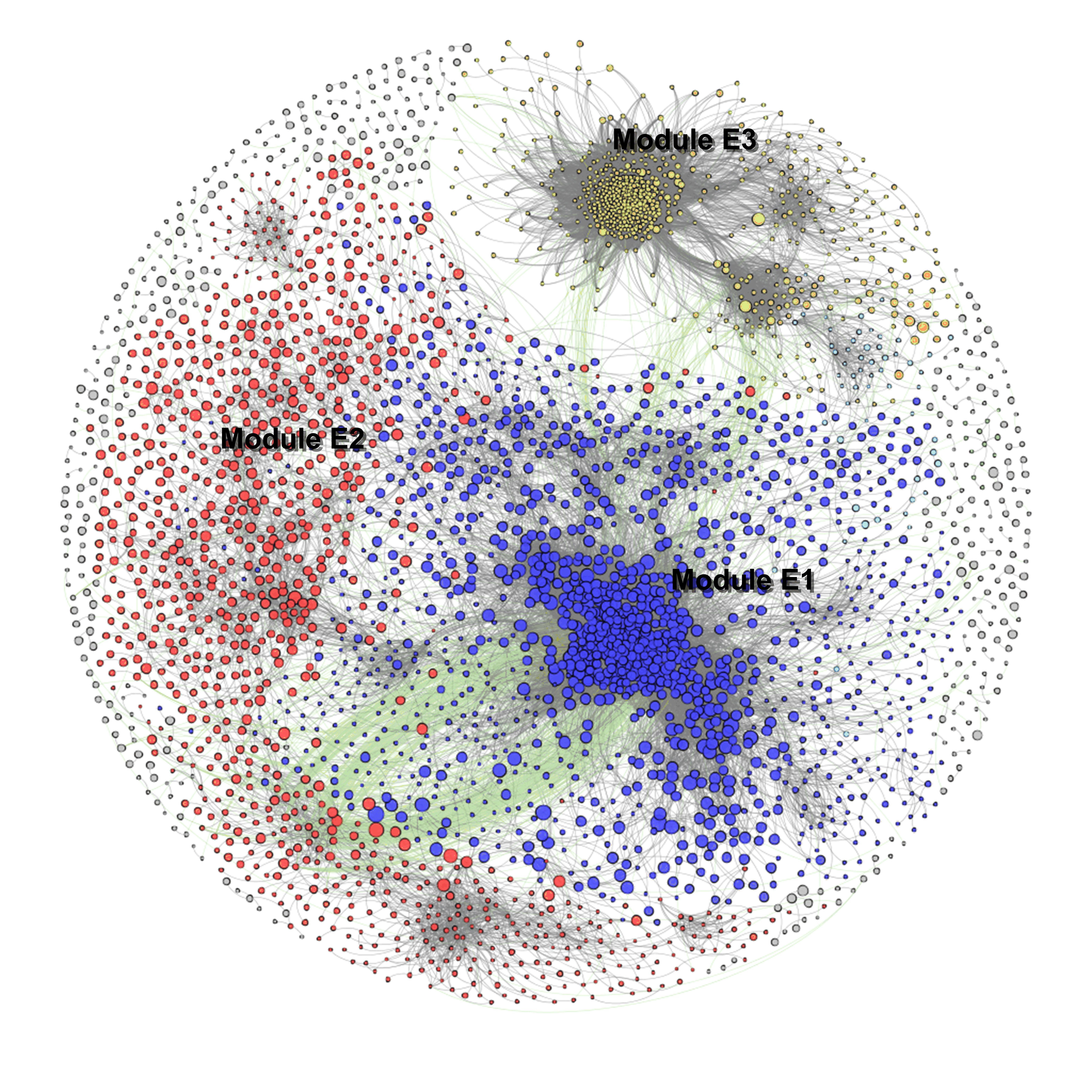

Supplement: FIG S6 [file msystems.01368-20-sf006.jpg]
