# Supplementary material for: Continental-Scale Paddy Soil Bacterial Community Structure, Function, and Biotic Interaction
Source: mSystems. 2021 Sep 21;6(5):e01368-20. doi: 10.1128/mSystems.01368-20 (PMC8547477; doi:10.1128/mSystems.01368-20)
Supplement: TABLE S1 [file msystems.01368-20-st001.docx]

**Table S1**

| Percentage, % | Continental | inter-regional | intra-regional | Sanjiang Plain | Lianghu Plain | Taihu Plain | Hani Terrace |
| --- | --- | --- | --- | --- | --- | --- | --- |
| betaNTI < -2 | 95.5 | 94.6 | 98.0 | 99.6 | 100.0 | 95.4 | 96.8 |
| \|betaNTI\| < 2 | 4.4 | 5.3 | 2.0 | 0.4 | 0.0 | 4.3 | 3.2 |
| betaNTI > +2 | 0.1 | 0.1 | 0.1 | 0.0 | 0.0 | 0.3 | 0.0 |
|  | Continental | inter-regional | intra-regional | Sanjiang Plain | Lianghu Plain | Taihu Plain | Hani Terrace |
| RC.bray < -0.95 | 7.0 | 5.2 | 12.7 | 8.0 | 12.3 | 17.2 | 12.6 |
| \|RC.bray\| < 0.95 | 23.4 | 12.3 | 55.0 | 89.1 | 51.7 | 46.8 | 32.8 |
| RC.bray > +0.95 | 69.4 | 81.4 | 32.1 | 2.9 | 36.0 | 36.0 | 54.2 |
