# Supplementary material for: Continental-Scale Paddy Soil Bacterial Community Structure, Function, and Biotic Interaction
Source: mSystems. 2021 Sep 21;6(5):e01368-20. doi: 10.1128/mSystems.01368-20 (PMC8547477; doi:10.1128/mSystems.01368-20)
Supplement: TABLE S2 [file msystems.01368-20-st002.docx]

**Table S2**

|  | Keystone taxa identified in paddy soil bacterial network | | | |
| --- | --- | --- | --- | --- |
| **ID** | **Module class** | **PageRank score** | **Relative abundance (%) in paddy soil** | **Taxonomy** |
| OTU1597 | P1 | 0.954 | 0.86 | Proteobacteria; Betaproteobacteria; DQ395705_o; EU786132_f; AF467301_g; EF126238_s |
| OTU686 | P1 | 0.912 | 0.68 | Chlorobi; Ignavibacteriae; GQ472436_o; GQ472436_f; GQ472436_g |
| OTU298 | P1 | 0.938 | 0.58 | Chlorobi; Ignavibacteriae; HQ014634_o; HQ014634_f; AY118151_g; AY118151_s |
| OTU714 | P1 | 1.000 | 0.34 | Acidobacteria; Solibacteres; Solibacterales; Solibacteraceae; EU589264_g; AM162424_s |
| OTU343 | P1 | 0.920 | 0.32 | Proteobacteria; Deltaproteobacteria; EU617842_o; EU617842_f; EU881182_g; HQ864112_s |
| OTU641 | P1 | 0.962 | 0.30 | Proteobacteria; Deltaproteobacteria; Desulfobacterales; HM243977_f; HM243977_g |
| OTU299 | P1 | 0.950 | 0.28 | Proteobacteria; Deltaproteobacteria; EU617842_o; FM253643_f; FM253643_g; FM253643_s |
| OTU688 | P1 | 0.912 | 0.11 | Gemmatimonadetes; GU568020_c; GU568020_o; GU568020_f; GU568020_g; EF540394_s |
| OTU22819 | P1 | 0.965 | 0.09 | Aminicenantes_OP8; AQSP_c; AQSP_o; AQSP_f; HQ183973_g; AB364867_s |
| OTU12122 | P1 | 1.000 | 0.05 | Chloroflexi; Dehalococcoidia; Dehalococcoidales; DQ811888_f; DQ811888_g; HM243964_s |
| OTU5 | P2 | 0.915 | 8.72 | Bacteroidetes; Bacteroidia; Bacteroidales; GU454901_f; GU454901_g; EU644258_s |
| OTU260 | P2 | 0.911 | 2.73 | Bacteroidetes; Bacteroidia; Bacteroidales; GU454901_f; GU454901_g; JN540128_s |
| OTU255 | P2 | 0.929 | 0.93 | Chloroflexi; Anaerolineae; Anaerolinaeles; Anaerolinaceae; GQ500701_g; AB240513_s |
| OTU7574 | P2 | 0.918 | 0.80 | Acidobacteria; HQ645210_c; HQ645210_o; HQ645210_f; HQ645210_g |
| OTU2587 | P2 | 1.000 | 0.06 | Chloroflexi; Anaerolineae; Anaerolinaeles; Anaerolinaceae; Anaerolinea |
